# A Dome-Shaped Aerosol Box for Protection During a Pandemic

**DOI:** 10.7759/cureus.22267

**Published:** 2022-02-16

**Authors:** Satoshi Ueno, Masahiro Iwabuchi, Reiko Suzuki, Hitoshi Izuha, Ken Iseki

**Affiliations:** 1 Emergency and Critical Care Medical Center, Fukushima Medical University Hospital, Fukushima, JPN; 2 Emergency Medicine, Fukushima Medical University, Fukushima, JPN; 3 Medical Engineering, Fukushima Medical University, Fukushima, JPN

**Keywords:** covid-19, stretcher type, bed type, aerosol boxes, a dome-shaped aerosol containment device with negative pressure (dawn)

## Abstract

With the rise of COVID-19, the use of aerosol boxes when interacting with COVID-19 patients has increased. However, their use has been controversial.

We have been involved in the development of a dome-shaped aerosol containment device with negative pressure (DAWN), an aerosol box that can maintain negative pressure inside at all times.

There are two types of DAWN: one is mounted on a bed (bed type) and the other is mounted on a stretcher (stretcher type). Each device has its own characteristics and can be selected depending on the situation. The bed type has enough space inside to allow procedures to be performed easily. The stretcher type can be attached to a stretcher and can maintain negative pressure when the patient is being moved. Due to the negative pressure structure and easy change of nonwoven fabric adopted in both types of DAWN, it is expected to prevent the scattering of aerosol when it is removed, which is a problem of conventional aerosol boxes.

DAWN will contribute to reducing the enormous psychological stress of medical personnel who treat infections, and will contribute to reducing aerosol dispersion.

## Introduction

COVID-19 infection has resulted in a global pandemic, starting after the first case was discovered in 2019, and at the time of writing continues to cause concern among health care providers (HCPs) in terms of healthcare disruption and secondary infection [1].

With the ongoing COVID-19 pandemic, the risk of the disease spreading via aerosol-generating procedures such as tracheal intubation and bronchoscopy is still high. When treating COVID-19, protection against HCPs infection must be thorough and prioritized, and aerosol-generating procedures should be avoided whenever possible. However, in emergency and intensive care, these procedures are often essential for patient treatment and survival, and any delay in their implementation would be a detriment to the patients. Since the beginning of the COVID-19 pandemic, there have been many reports of the use of aerosol boxes, with the aim being to reduce aerosol dispersion, thereby preventing secondary infections among HCPs [2-4].

The use of aerosol boxes is a result of a shortage of personal protective equipment (PPE), as well as a desire on the part of HCPs to lower the risk of infection when treating the patient. However, the use of aerosol boxes is controversial.

Before a new medical device is used clinically, its safety and efficacy must be assessed. In the case of aerosol boxes, the ability to protect against infection while not interfering with the performance of the procedure being performed is crucial. However, it has been pointed out that aerosol boxes themselves can spread aerosols [5,6]. This is a serious problem with regard to virus dispersion. In addition, there have been reports of extended procedure times due to the limited working space inside the box [7-9]. This can lead to adverse effects on patients.

A dome-shaped aerosol containment device with negative pressure (DAWN), a new device that could solve these problems, has been developed by a company in Minamisoma City, Fukushima Prefecture, Japan. In 2011, Fukushima Prefecture experienced a nuclear accident after the Great East Japan Earthquake. The experience of dealing with radioactive dust at that time was utilized in the manufacturing and development of DAWN. Fukushima Medical University Hospital, as a co-developer, played a role in the development of the device by providing advice.

## Technical report

There are two types of DAWN: bed type and stretcher type. Both types commonly have a transparent dome with a suction port connected to a negative pressure aspirator, which maintains a negative pressure of less than -2.5 Pa inside the dome at all times. The capacity of the exhaust unit is 500 L/min, which allows the air inside the dome to be replaced 2-3 times/min in the bed type and about 9 times/min in the stretcher type, preventing the accumulation of aerosols in the dome. The exhaust unit is also equipped with a high-efficiency particulate air (HEPA) filter to prevent splashing and leakage. The DAWN is covered with a nonwoven fabric cover with high viral barrier properties. This cover can be easily replaced for each patient, simplifying the disinfection of the dome frame.

**Figure 1:**
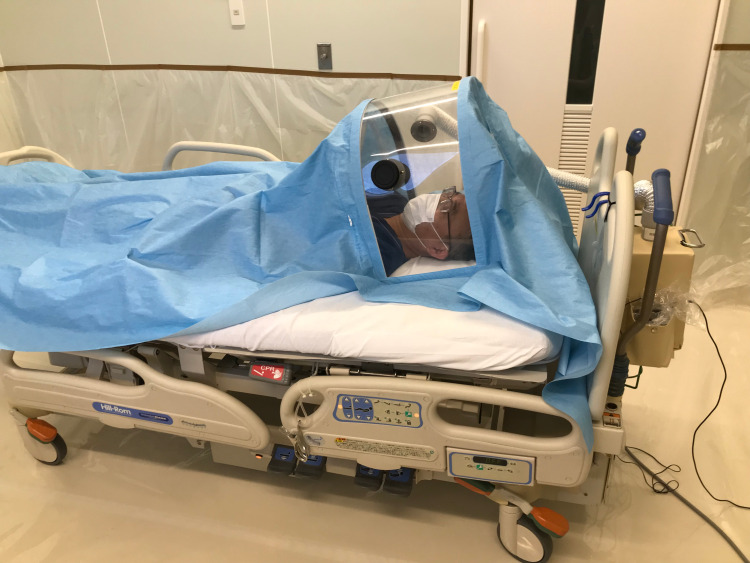
The bed-type DAWN

Next, we will discuss the features of the bed type DAWN (Figure 1). The interior of the bed type DAWN is large (500 mm long × 1400 mm wide × 550 mm high) enough to allow aerosol-generating procedures to be performed comfortably. In addition, the dome has two retractable holes, which allow an assistant to pass tools to the proceduralist. Therefore, it is expected that the procedure will not be delayed by the limited working space. In addition, the bed-type DAWN can be used on a bed at all times, and because of the large space inside the dome, it can be used for long periods of time, causing minimal stress to the patient.

**Figure 2:**
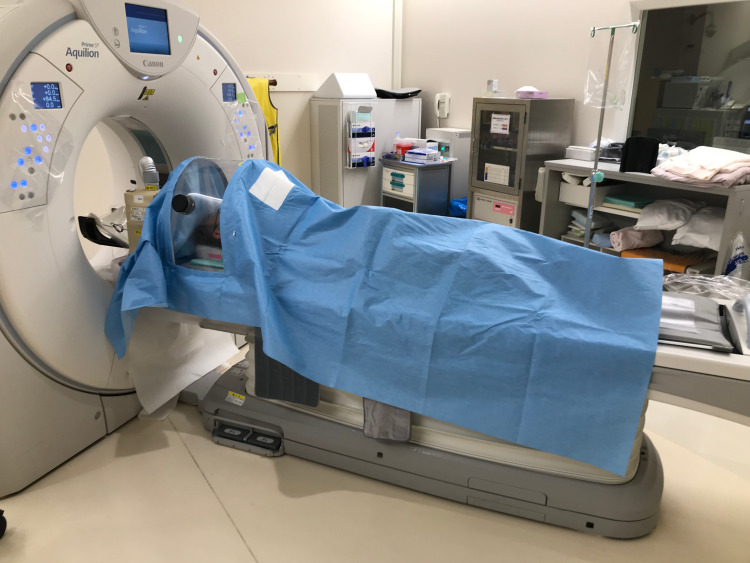
The stretcher-type DAWN

The stretcher type DAWN is smaller than the bed type (300 mm long × 800 mm wide × 450 mm high), and can be used when patients are being transported (Figure 2). By using portable dry-cell batteries, the DAWN-mounted stretcher can be moved while maintaining negative pressure inside the dome, thus preventing splashing during transportation.

In addition, the size of the stretcher type DAWN is small enough to pass through the gantry of a general computed tomography (CT) system, and depending on the scanning area, it is possible to undergo CT without removing DAWN. Although the stretcher type DAWN is more compact than the bed type, it is sufficiently spacious compared to the conventional aerosol box, and is unlikely to limit the working space for emergency tracheal intubation in emergency rooms.

## Discussion

DAWN has three strengths. First, negative pressure can be maintained inside the dome. To date, there have been no reports of a negative pressure aerosol box equipped with a HEPA filter. We believe that the ability to maintain negative pressure inside the dome at all times will reduce the burden of procedures on HCPs and prevent secondary infections more than the conventional aerosol box when performing aerosol-generating procedures.

Secondly, delay of procedures due to limited working space has been a problem of conventional aerosol boxes. However, the inside of DAWN has a large amount of space, makings it possible to perform the necessary procedure without delay. Furthermore, we believe that procedures performed in a negative pressure environment with sufficient working space will contribute to reducing the psychological burden on proceduralists, as well as a reduction of procedure failure.

Third, the nonwoven fabric covers used on the DAWN are easy to install and remove. One of the problems with conventional aerosol boxes is the scattering of viruses and particles when such covers are installed and removed. In DAWN, as the negative pressure is maintained at all times, minimizing aerosol accumulation in the dome, aerosols are unlikely to be scattered during the installation and removal of the covers.

DAWN with these features is also expected to reduce the psychological burden on HCPs who treat infected patients. One of the problems with aerosol boxes is the creation of a false sense of security in the minds of HCPs. That is, despite the use of a device that may delay the procedure and spread aerosols, the user may place too much confidence in the device and may be harmed by its use. However, there is still a concern that this problem may occur in the use of DAWN; therefore, its effectiveness needs to be demonstrated in the future. On the other hand, because of the large space available and the portable nature of DAWN, further expansion of its use is being considered.

Above all, as medical professionals who have been working clinically under a great deal of stress due to the COVID-19 pandemic, we hope that the use of DAWN will contribute to the dawn of the end of COVID-19 because of its effectiveness in overwhelmingly reducing the psychological burden on the part of HCPs.

## Conclusions

Unlike conventional aerosol boxes, the use of DAWN is not expected to increase the time required to perform aerosol-generating procedures. In addition, we believe that the use of DAWN will reduce the psychological burden on HCPs, and will reduce aerosol dispersion.
